# Bioactivity and genotoxicity effects of certain compounds on *Theba pisana* and *Monacha cartusiana* land snails

**DOI:** 10.1038/s41598-025-22221-w

**Published:** 2025-10-27

**Authors:** Nada M. T. Abbas, Sahar I. M. Abd El-Wahed, Hesham A. M. Ibrahim, Mona F. A. El-Sitiny, Mohammed G. Mahmoud

**Affiliations:** 1 Department of Harmful Animals, Plant Protection Research Institute, Agricultural Research Center, Dokki, Giza, 12619 Egypt; 2 Department of Agricultural Zoology and Nematology, Faculty of Agriculture, Assiut Branch, Al Azhar University, Assiut, 71524 Egypt; 3 Plant Protection Department, Agricultural Faculty, Zagazig University, Zagazig, Egypt

**Keywords:** *Theba pisana*, *Monacha cartusiana*, Molluscicidal activity, RAPD-PCR, Genotoxicity, Genetic polymorphism, Zoology, Molecular biology

## Abstract

*Theba pisana* and *Monacha cartusiana*, both land snails, are agricultural pests that have caused significant losses in many orchards and fields in various areas of Egypt. In this study, the efficacy of nicotinamide, imidacloprid, and silver nitrate nanoparticles compounds compared to oxamyl were evaluated against these two snail species using residual film and poison bait techniques under laboratory and field conditions to identify alternative, more effective options to conventional pesticides. In addition, the genotoxicity impacts of these compounds on the tested snails were thoroughly investigated. Results showed that, for *T. pisana*, oxamyl exhibited the highest mortality rate, followed by nicotinamide and silver nitrate, with calculated LC_50_ values of 0.028%, 0.049%, and 0.120% for residual film technique and 0.546%, 0.971%, and 1.333% for poison bait technique. Similarly, for *M. cartusiana*, oxamyl exhibited the highest toxicity, followed by nicotinamide and silver nitrate, with LC_50_ values of 0.054%, 0.063 and 0.103% in residual film method, While in the poison bait method, oxamyl showed the highest toxicity, followed by silver nitrate and nicotinamide with LC_50_ values of 0.273%, 1.150 and 1.204, respectively. Imidacloprid showed the lowest efficacy in both species with LC_50_ values of (0.19, 0.14%) and (1.57, 1.48%) for residual film and baits techniques, respectively. The field evaluation of tested compounds against *T. pisana* and *M. cartusiana* snails using poisonous baits revealed varying effectiveness in reducing snail populations. Oxamyl showed the highest reduction rates, with 46.31%, 63.63%, and 90.85% for *T. pisana* and 29.47%, 63.92%, and 92.13% for *M. cartusiana* after 1, 7, and 21 days of treatment, respectively. Nicotinamide and silver nitrate nanoparticles were also effective, reducing populations by 81.71% and 72.83% for *T. pisana* and 92.62% and 84.95% for *M. cartusiana* after 21 days. Imidacloprid compound resulted in the lowest reductions, with 60.64 and 63.26% for two species, respectively. Respecting molecular diagnosis RAPD- PCR, the results demonstrated that the presence of polymorphism in the two types of snails treated with tested compounds.

## Introduction

 Gastropods play vital ecological roles as agricultural pests, intermediate hosts, food sources, and bioindicators of environmental quality^1^. These organisms are also valuable for biomonitoring contaminated areas and informing soil management strategies based on ecotoxicological risks through bioassays^2^. Terrestrial gastropods have become important economic pests attacking various vegetation in Egypt^3^, because they damage numerous agronomic, horticultural, and ornamental plants^4^. The severity of the damage inflicted by snails depends not only on their activity and population density, but also on their feeding habits, which differ from one species to another^5^, which can further lead to contamination through their bodies, feces, and mud^6^. Recent reports highlight that terrestrial gastropods are emerging invasive pests in several Mediterranean and Middle Eastern countries, causing measurable yield losses in cereals, vegetables, and orchards^7,8^. *Theba pisana*, a species common across the Mediterranean basin, thrives in diverse environments, including dune and also near rivers habitats, and is identified by its robust, whitish shell with brown bands, with a diameter of 15–20 mm and a height of 10–15 mm^9^. This snail’s small size, high survival rate, and prolific reproduction make it a particularly problematic pest^10^. The clover snail, *Monacha cartusiana*, is one of the most economically damaging animal pests, spreading throughout Egypt and severely harming its agriculture^11^. Recent field surveys in Egypt confirm that *T. pisana* and *M. cartusiana* dominate land-snail communities in citrus and winter crops and can reach damaging densities, with abundance patterns strongly shaped by season and canopy level^12,13^.

Usually, in pest control programs, traditional pesticides are relied upon primarily, despite the problems they cause. However, it is necessary to search for more effective, anti-resistant compounds to use in controlling these pests. The overuse of conventional molluscicides such as methomyl and metaldehyde has raised serious concerns due to resistance development, environmental contamination, and risks to non-target organisms, highlighting the urgent need for safer alternatives^14,15^.Nicotinamide (NAM) has been shown to alter behavior in *Caenorhabditis elegans* and *Drosophila*, serving as an agonist of TRPV channels affecting sensory neurons and mimicking the mode of action of insecticides used to control phloem-feeding insects^16^. Oxamyl, a systemic and contact pesticide, inhibits acetylcholinesterase (AChE), which hydrolyzes the excitatory neurotransmitter acetylcholine at the neuromuscular synapse^17^. Imidacloprid is a member of the chloronicotinyl class. The mode of action of imidacloprid is to block neurotransmission by postsynaptic antagonism of nicotinic acetylcholine receptors^18^. It has a high selectivity towards the site of action within insects together with the high safety margin on mammals^19^. Silver nanoparticles (AgNPs) are one of the most important classes and most commonly used nanomaterials, silver nanoparticles toxic effects include cytotoxicity via apoptosis and necrosis, lethality, oxidative stress, DNA and cell membrane damage, mitochondrial malfunction, inflammation and decreased cellularproliferation^20^.

Genotoxicity is defined as the capability of a substance or agent to disrupt the structure, informational content, or organization of DNA, though it is not always linked to mutagenicity. Many soil pollutants have genotoxic effects^21,22^, and can lead to the collapse of natural populations, notably molluscs^23–26^. Furthermore, in vivo studies are necessary when in vitro assessments indicate a positive genotoxicity potential^27^. Despite the recognized importance of these pests, limited studies have examined the combined bioactivity and genotoxicity of alternative compounds, particularly nicotinamide and silver nanoparticles, against *T. pisana* and *M. cartusiana* under both laboratory and field conditions. Addressing this gap is essential to develop eco-friendly strategies for sustainable snail management^28,29^. This study aims to evaluate the toxicity of certain compounds on *T. pisana*,* M. cartusiana* using some molecular measurements to better understand their impacts and inform pest management strategies.

## Results

### Bioactivity under laboratory conditions

#### Molluscicidal activity of certain compounds against *Theba pisana snails* using two techniques under laboratory conditions

The effect of nicotinamide, imidacloprid, and silver nitrate nanoparticles compounds in comparison to oxamyl as a reference pesticide against *Theba pisana* was evaluated using Residual Film and Poison Bait techniques, as shown in Table 1. The results revealed that oxamyl exhibited the highest mortality rate, followed by nicotinamide, outperforming the other compounds. The calculated LC_50_ values for the Residual Film technique were 0.028%, 0.049%, and 0.120%, with corresponding toxicity indexes of 100%, 57.14%, and 23.33% for oxamyl, nicotinamide, and silver nitrate, respectively. Moreover, snail mortality increased with rising concentrations in all treatments. For the Poison Bait technique, the LC_50_ values were 0.546% and 0.971%, with toxicity indexes of 100% and 56.23% for oxamyl and nicotinamide, respectively.

**Table 1:** Efficacy of certain compounds compared to oxamyl as a reference compound against *Theba pisana snails* using two techniques under laboratory conditions.

Used technique	Compounds	LC_50_ (%)	95% confidence limits (lower–upper)	Regression equation	TI
Residual film	Nicotinamide	0.049	0.031–0.077	y = 1.856x + 7.436	57.14
	Imidacloprid	0.190	0.149–0.242	y = 3.608x + 7.642	14.73
	Silver nitrate nanoparticles	0.120	0.091–0.159	y = 3.108x + 7.872	23.33
	Oxamyl	0.028	0.013–0.058	y = 1.161x + 6.807	100.0
Poison baits	Nicotinamide	0.971	0.776–1.214	y = 3.988x + 5.054	56.23
	Imidacloprid	1.574	1.421–1.743	y = 8.456x + 3.336	34.68
	Silver nitrate nanoparticles	1.333	1.174–1.514	y = 6.746x + 4.155	40.96
	Oxamyl	0.546	0.342–0.871	y = 2.269x + 5.752	100.0

LC_50_: 50% lethal concentration.

TI , Toxicity index.

#### Molluscicidal activity of certain compounds against *Monacha cartusiana* snails using two techniques under laboratory conditions

The biological activities of nicotinamide, imidacloprid, and silver nitrate nanoparticles compounds, in comparison to oxamyl as a reference pesticide, against the terrestrial snail *Monacha cartusiana* is detailed in Table 2. Under laboratory conditions, two techniques were evaluated. For the residual film technique, oxamyl demonstrated the highest toxicity, followed by nicotinamide, with LC_50_ values of 0.054% and 0.063%, and toxicity index values of 100% and 85.71%, respectively. Similarly, using the poison bait method, oxamyl showed the greatest toxic effects, followed by silver nitrate, with LC_50_ values of 0.273% and 1.150%, and toxicity index valuesof 100% and 23.73%, respectively. In contrast, imidacloprid exhibited the lowest efficacy, with LC_50_ values of 0.140% and 1.480% for the residual film and poison bait methods, respectively. Based on the results obtained, the contact method showed higher effectiveness than the poison bait method against the tested snails under laboratory conditions.

### The applied field tests

After field evaluation of the tested compounds against the two species of snails, *T. pisana* and *M. cartusiana*, using poisonous baits technique, the results in Table 3 showed that the effect of the tested pesticides in reducing the numbers of these snails showed different degrees of reduction compared to the control plots.

**Table 2:** Efficacy of certain compounds compared to oxamyl as a reference compound against *Monacha Cantiana* snails using two techniques under laboratory conditions.

Used technique	Compounds	LC_50_ (%)	95% Confidence limits (lower–upper)	Regression equation	TI
Residual film	Nicotinamide	0.063	0.041–0.097	y = 2.019x + 7.435	85.71
	Imidacloprid	0.140	0.123–0.160	y = 6.879x + 10.858	38.57
	Silver nitrate nanoparticles	0.103	0.090–0.117	y = 6.903x + 11.804	52.42
	Oxamyl	0.054	0.033–0.091	y = 1.618x + 7.039	100.0
Poison baits	Nicotinamide	1.204	1.062–1.364	y = 6.987x + 4.452	22.67
	Imidacloprid	1.480	1.313–1.669	y = 7.085x + 3.792	18.44
	Silver nitrate nanoparticles	1.150	0.953–1.387	y = 4.443x + 4.730	23.73
	Oxamyl	0.273	0.165–0.452	y = 1.893x + 6.066	100.0

LC_50_: 50% lethal concentration.

TI , Toxicity index.

It was found that oxamyl compound gave the highest reduction rate with values (46.31, 63.63 and 90.85%) and (29.47, 63.92 and 92.13%) after 1, 7 and 21 days of treatment for both species of *T. pisana* and *M. cartusiana*, respectively, followed in effectiveness under field conditions by nicotinamide and silver nitrate nanoparticles compounds with values ​​(81.71 and 72.83%), (92.62 and 84.95%) after 21 days of treatment for *T. pisana* and *M. cartusiana* snails, respectively, while imidacloprid compound gave the lowest reduction rates with values ​​(31.9 and 60.64%), (48.19 and 63.26%) after 7 and 21 days of treatment for *T. pisana* and *M. cartusiana*, respectively. From the obtained results, we find that the decrease rates gradually increase during the exposure periods from the 1st day until the 21st day.

**Table 3:** Reduction in *Theba Pisana* and *Monacha cartusiana* snail populations after exposed to toxic baits with tested toxicants in field conditions at Sidi Salem, Kafr El-Sheikh Governorate.

Treatments	Reduction percentages after treatment/days						General mean
	1-days	3-days	7-days	10-days	15-days	21-days	
*Theba pisana*							
Nicotinamide	11.63	53.07	61.56	74.23	78.45	81.71	60.11
Imidacloprid	12.25	15.18	31.9	42.71	55.85	60.64	36.42
Silver nitrate nanoparticles	37.15	39.94	53.2	59.76	68.35	72.83	55.21
Oxamyl	46.31	53.64	63.63	75.17	81.79	90.85	68.57
*Monacha cartusiana*							
Nicotinamide	14.07	29.7	50.1	66.56	82.39	92.62	55.91
Imidacloprid	28.08	38.86	48.19	55.95	58.34	63.26	48.78
Silver nitrate nanoparticles	17.17	32.71	55.42	65.98	78.97	84.95	55.87
Oxamyl	29.47	53.22	63.92	73.38	80.42	92.13	65.42

### Moecular diagnosis (RAPD-PCR)

**Fig. 1:**
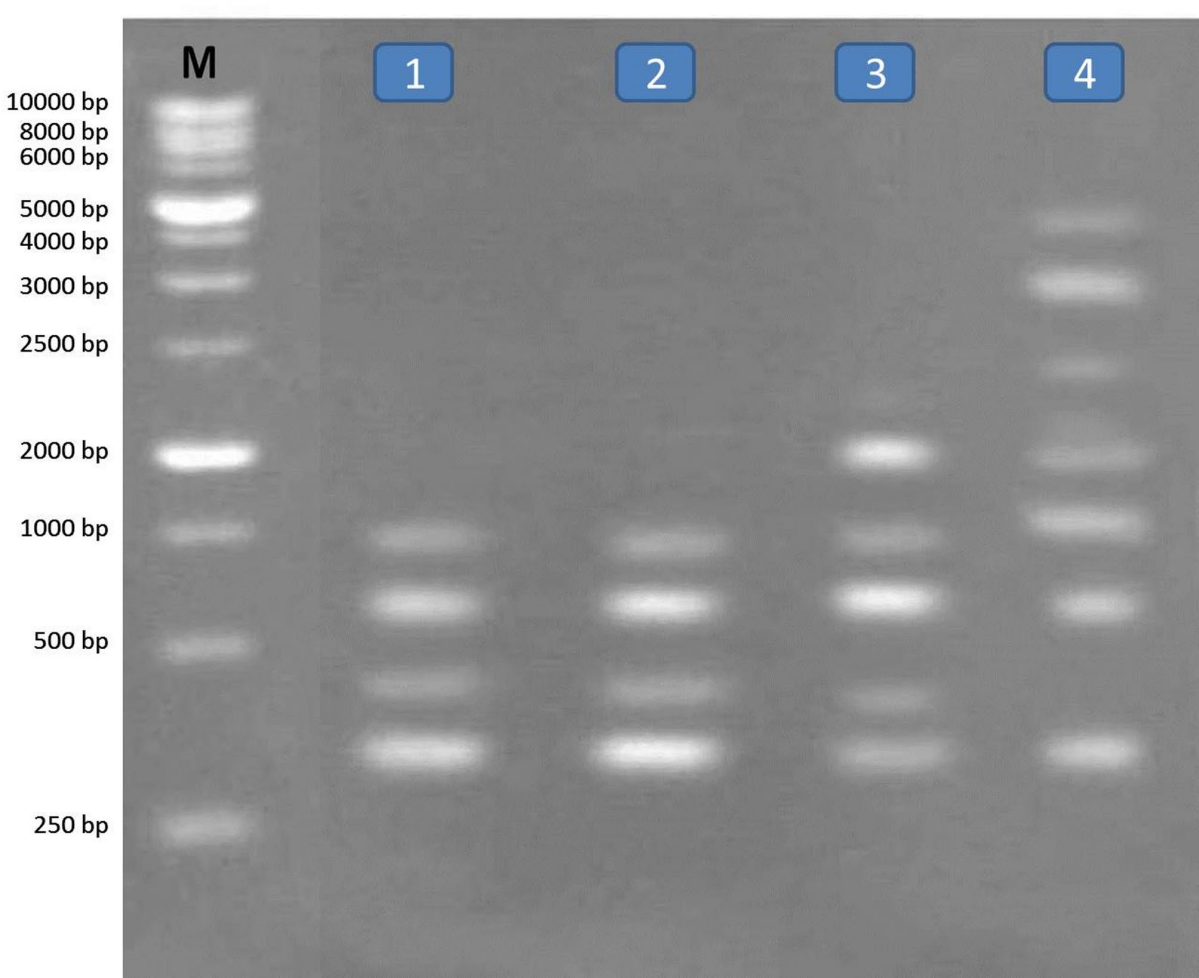
RAPD- PCR DNA with primer (1) for Lane M: Marker, L1: *Monacha cartusiana* treated with with imidacloprid L2: *M. cartusiana* treated with silver nitrate, L3: *M. cartusiana* treated with nicotinamide and L4: *M. cartusiana* treated with oxamyl.

**Fig. 2:**
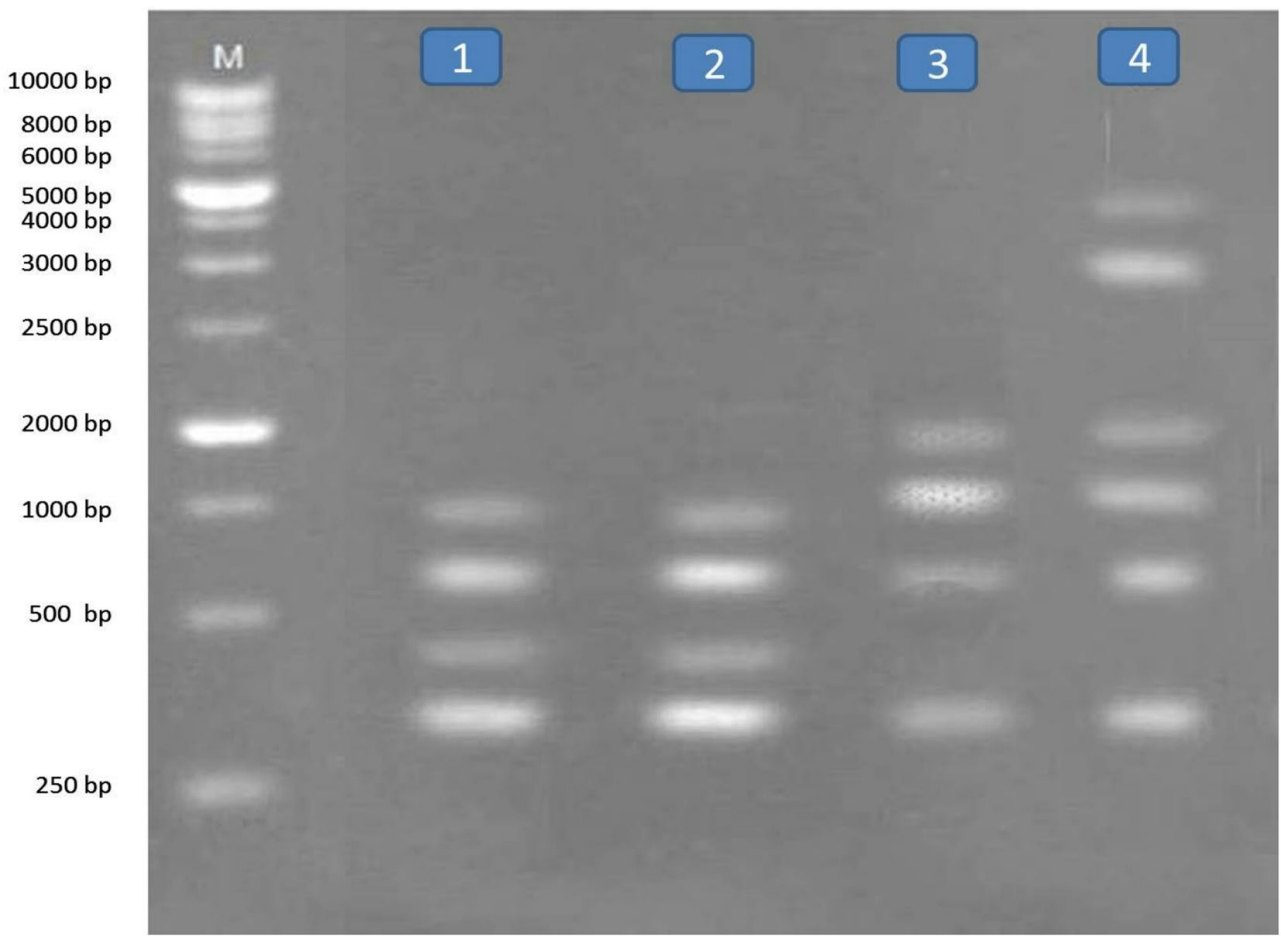
RAPD- PCR DNA with primer (1) for Lane M: Marker, L1: *Theba pisana* treated with imidacloprid L2: *T. pisana* treated with silver nitrate, L3: *T. pisana* treated with nicotinamide and L4: *T. pisana* treated with oxamyl.

**Fig. 3:**
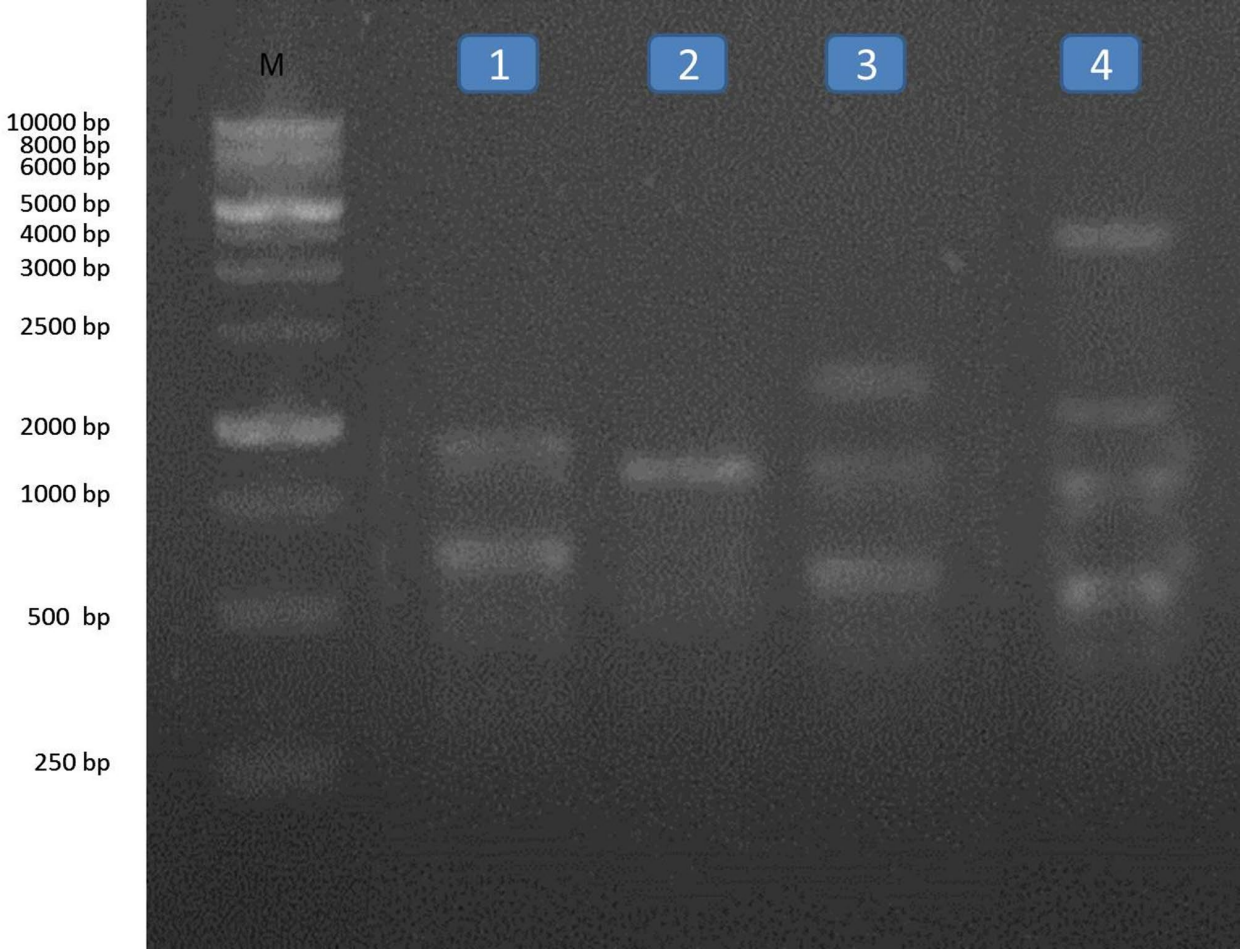
RAPD- PCR DNA with primer (2) for Lane M: Marker, L1: *Monacha cartusiana* treated with imidacloprid L2: *M. cartusiana* treated with silver nitrate, L3: *M. cartusiana* treated with nicotinamide and L4: *M. cartusiana* treated with oxamyl.

**Fig. 4:**
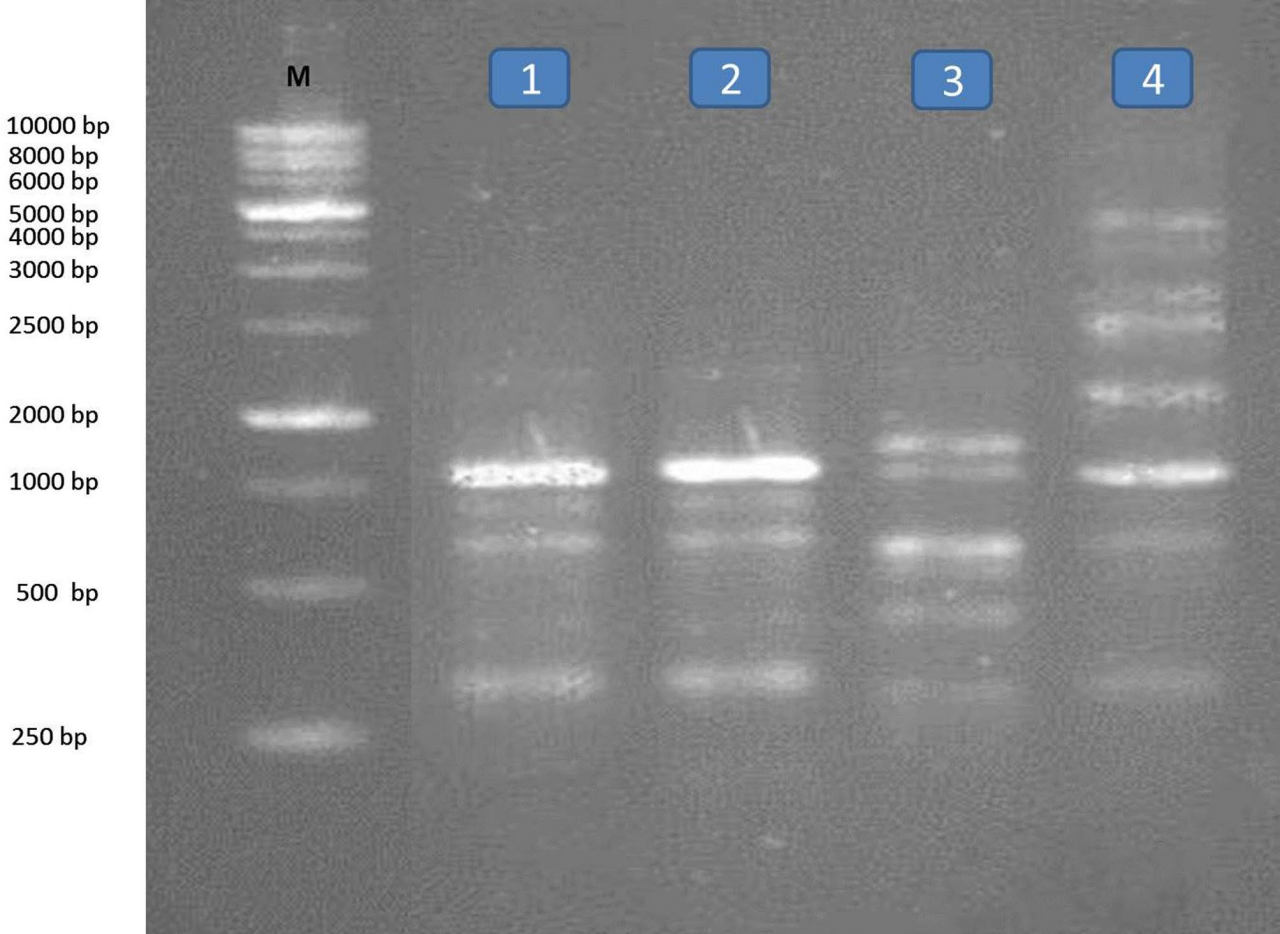
RAPD- PCR DNA with primer (2) for Lane M: Marker, L1: *Theba pisana* treated with imidacloprid L2: *T. pisana* treated with silver nitrate, L3: *T. pisana* treated with nicotinamide and L4: *T. pisana* treated with oxamyl.

RAPD analysis method is a simple and very sensitive method and appears effective in detecting genetic variations among the two different snails treated with five compounds belonging to different groups. The molecular weight of fragments after RAPD-PCR reaction with the two primers in the two types of snails were separated, range of fragment size, the total number of fragments, number of monomorphic fragments, number of polymorphic fragments and percentage of polymorphism obtained per RAPD shown in Tables 4 and 5 and Figs. 1, 2, 3 and 4. A total number of amplified fragments were 18.0 with average 9.0 fragments per primer 1 which ranged from 250 to 5000 bp approximately. The total polymorphism percentage was 72.50%, where Primer 2 revealed the highest percentage of polymorphism (77.5%). The total polymorphic fragments were 13.0 with average 6.50 fragments. The two tested snails treated with oxamyl recorded the highest value of amplified fragments 13 with primer 1 and 10 with primer 2 ranged from 250 to 5000 bp across all the two primers, followed by nicotinamide, silver nitrate nanoparticles and imidacloprid recorded values of amplified fragment (9.0, 8.0 and 8.0; 6.0, 4.0 and 5.0 with primer 1 and 2, respectively) which ranged from 250 to 5000 bp.

**Table 4:** The polymorphism in fragment size after RAPD-PCR reaction with primer 1 in two species of *snails* (*Theba Pisana* and *Monacha cartusiana*) treated with different five compounds.

Type	Range of fragment size	1	2	3	4	Total no. of fragments	Monomorphic fragments	Polymorphic fragments	Polymorphism %
*Theba pisana*	250–5000 bp	4	4	4	6	8	2	6	75%
*Monacha cartusiana*	250–5000 bp	4	4	5	7	10	3	7	70%
Total	250–5000 bp	8	8	9	13	18	5	13	72.5%
Average	–	4.0	4.0	4.5	6.5	9.0	2.5	6.5	

**Table 5:** The polymorphism in fragment size after RAPD-PCR reaction with primer 2 in two species of snails (*Theba Pisana* and *Monacha cartusiana*) treated with different five compounds.

Type	Range of fragment size	1	2	3	4	Total no. of fragments	Monomorphic fragments	Polymorphic fragments	Polymorphism %
*Theba pisana*	250–4000 bp	3	3	3	6	8	2	6	75%
*Monacha cartusiana*	250–5000 bp	2	1	3	4	5	1	4	80%
Total	250–5000 bp	5	4	6	10	13	3	10	77.5%
Average	–	2.5	2.0	3.0	5.0	6.5	1.5	5.0	

## Discussion

The results obtained showed that the compounds nicotinamide, imidacloprid and silver nitrate nanoparticles have an effective molluscicide activity compared to oxamyl as a reference compound, which gave the highest activity against the snails *Theba pisana* and *Monacha obstructa*. In line with what Shaker et al.^30^. found, oxamyl and methomyl were the most effective molluscicides against the brown snail *Eobania vermiculata*, with LC_50_ values of 0.004 and 0.024%, respectively, after 96 h of treatment in the laboratory. Aioub et al.^31^ and Hemmaid and Mohammadein^32^ also reported that the carbamate oxamyl compound showed high efficiency as a molluscicide against land snails. Mohamed and Morsy^33^ applied nanosilica to control *E.vermiculata* adults. Populations of *E. vermiculata* were decreased by the nanosilica. According to Attia et al.^34^, the hydrophilic nanosilica’s LC_50_ and LC_90_ for *Biomphalaria alexandrina* were 590 ppm/6 h and 980 ppm/48 h, respectively. For the freshwater snail *Pila virens*, the median lethal concentration of SiNPs after 96 h is 366.92 µg/L^35^. In field conditions, silica nanoparticles were the most effective treatment for controlling land snails *Monacha cantiana*, with a mean general reduction percent of 90.73%. Insecticide treatment came in second, with a mean general reduction percent of 87.46%^36^. Also, Hussein and Sabry^37^, reported that the recommended field rate of imidacloprid sharply decreased the hatchability percentage for the land snail, *E. vermiculata* to 16.2%, respectively, compared with 96.3% in control, highlighting its potential as a molluscicidal agent .

The present study revealed clear differences in the genotoxic effects of five tested compounds on two species of snails, with Oxamyl showing the strongest genotoxicity based on the highest polymorphism rates observed through RAPD-PCR analysis. This was followed by nicotinamide and silver nitrate nanoparticles, while imidacloprid exhibited the weakest genotoxic effect. These findings align with previous research highlighting the genotoxic potential of different pesticide formulations and their varying impacts on non-target organisms. Our results support the effectiveness of RAPD-PCR as a sensitive molecular tool to detect DNA alterations caused by environmental contaminants. Similar findings were reported by Baurand et al.^38^, who applied RAPD-PCR to detect genomic alterations in snail embryos exposed to commercial pesticide formulations such as Roundup^®^ and Corail^®^. Their study demonstrated band pattern changes indicative of DNA damage, including strand breaks, mutations, or chromosomal rearrangements. Notably, they observed the disappearance and appearance of RAPD bands, consistent with our findings of polymorphic fragments across the treated snail groups, particularly with Oxamyl and nicotinamide exposure. The study emphasized that RAPD-PCR is suitable for non-model organisms, such as land snails, and can be a powerful tool in ecotoxicogenomic assessments^29^.

In addation, a systematic review on engineered nanomaterials confirmed that silver nanoparticles induce oxidative stress, immunotoxicity, and genotoxicity in land snails, including *T*. *pisana*, via reactive oxygen species generation and DNA damage mechanisms^39^. Furthermore, the present findings align with the results obtained by Abd-El-Haleem et al.^40^, who investigated the genotoxicity of several pesticides, including methomyl, avaunt, pestban, and herbazed, on *M. cartusiana* using the comet assay. Their data revealed significant DNA damage indicators, such as increased tail length and tail moment, particularly in methomyl-treated snails. These results confirm the DNA-damaging capabilities of commonly used pesticides at sublethal concentrations and support the relevance of using both molecular (RAPD-PCR) and cellular (comet assay) techniques to evaluate genotoxicity in terrestrial mollusks. Moreover, their study, similar to ours, highlights the relatively lower genotoxic impact of chlorpyrifos compared to other tested compounds, further validating our results^40^. Moreover, a recent mini-review reinforced the value of land snails as indicators in genotoxicity studies, noting that RAPD-PCR, comet assays, and other biomarkers have effectively revealed DNA damage in snails exposed to metals, nanoparticles, and pesticides, including *T*. *pisana*, underscoring the methodological relevance of our approach^41^. In conclusion, the data presented here provide additional evidence of the varying genotoxic potential of different pesticides on non-target organisms. Our findings not only demonstrate the relative severity of genetic alterations induced by Oxamyl and other compounds but also highlight the utility of RAPD-PCR as a practical molecular tool for environmental biomonitoring. Future studies integrating molecular, cellular, and biochemical markers are encouraged to better understand the mechanisms of pesticide-induced genotoxicity and to inform regulatory policies.

## Materials and methods

### Experimental snails

Adults of the white garden snail, *Theba pisana* (O.F. Müller, 1774), and the glassy clover snail, *Monacha cartusiana* (O.F. Müller, 1774), of similar age and size were collected for laboratory testing. The white garden snails were gathered from infested lemon orchards, and the glassy clover snails came from heavily infested clover fields in Motobas district, Kafr El-Sheikh Governorate. Species identification followed Ali and Ramdane^42^. The snails were brought to the laboratory and placed in plastic containers filled with moist, sterilized sandy loamy soil maintained at 25 ± 2 °C and 75 ± 5% soil moisture. They were fed fresh lettuce leaves (*Lactuca sativa* L.) for 14 days to acclimate them to lab conditions, with unhealthy or dead snails removed to ensure only healthy specimens were used in the experiments^43^. After adaptation, snails were starved for the final 24 h before treatment^44^.

### Compounds used in bioassays

In both laboratory and field experiments, four different compounds were tested: Nicotinamide (pyridine-3-carboxamide) with the molecular formula of C_6_H_6_N_2_O), produced by Adwek company and manufactured by El Nasr company; oxamyl (Vydate 24% SL with a molecular formula of C7H13N3O3S), produced by Syngenta company and manufactured by KZ Pesticides & Chemicals company; imidacloprid (Imidastar 24% WP with a molecular formula of C_9_H_10_ClN_5_O_2_), produced by Sumitiomo Chemical India and manufactured by Elhelb Pesticides and Chemicals Company and silver nanoparticles.

### Preparation and characterization of silver nanoparticles

**Fig. 5:**
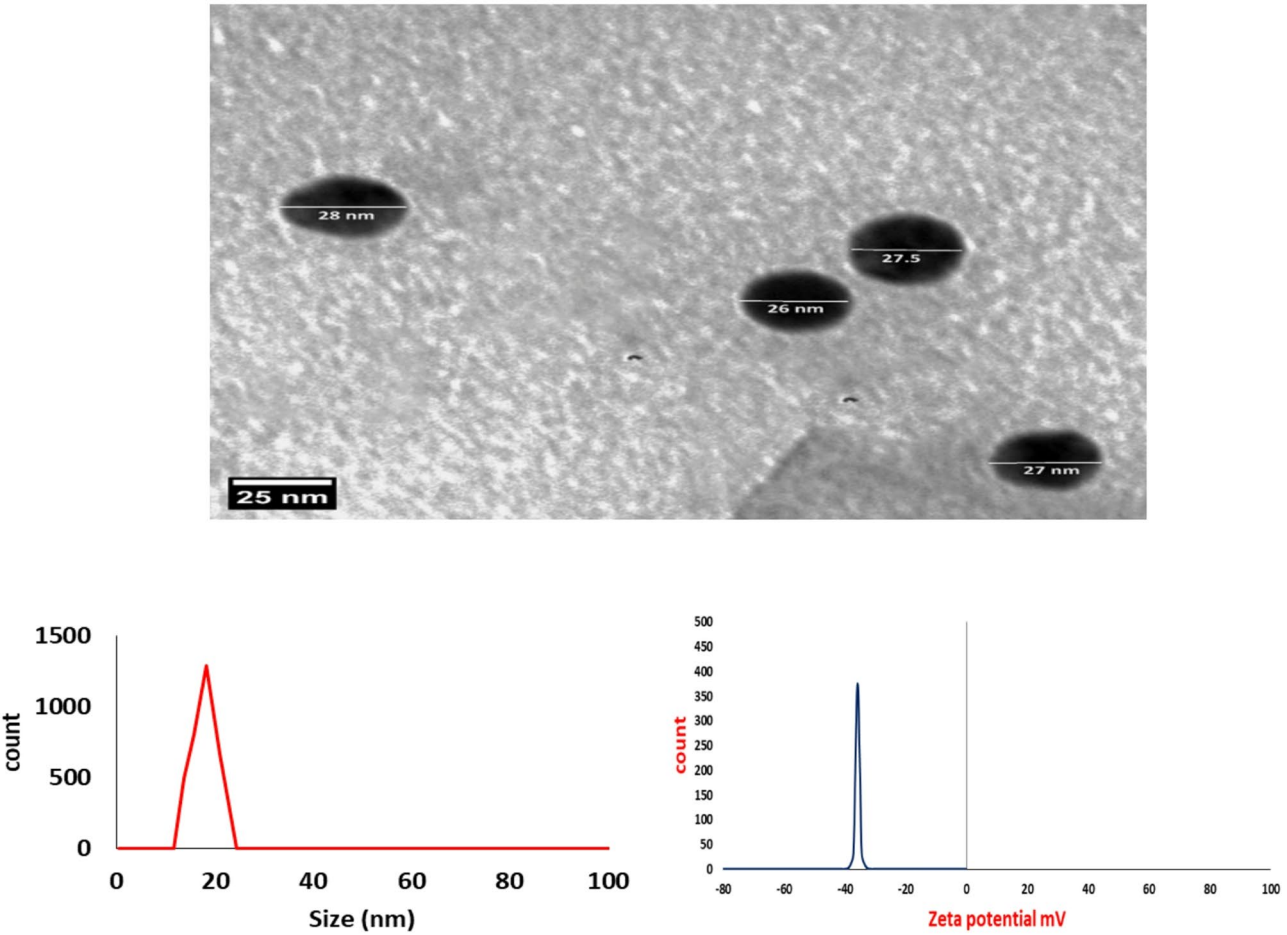
Characterization of silver nanoparticles using transmission electron microscopy (TEM), Zeta potential, and Zeta particles sizer.

Silver nanoparticles were characterized by different methods to analyze their particle sizes and aggregation states. A dynamic light scattering technique was performed to understand the stability and size distribution of the prepared silver nanoparticles. A TEM (transmission electron microscope) analysis showed that the silver nanoparticles were widely dispersed and composed of particles with an average of < 100 nm. Moreover, the TEM micrograph showed that the silver nanoparticles had a spherical morphology with an average size between (26–28 nm). The zeta potential results for the silver nanoparticles showed a negative surface charge with a value of approximately − 35 mV. The analysis showed that the size distribution range was within 20 nm and the curve showed a monomodal distribution (polydispersity index PDI) (Fig. 5).

### Bioactivity assays

#### Laboratory tests

Laboratory tests were conducted to assess the toxicity of specific compounds as molluscicides against two snail species, *T. pisana* and *M. cartusiana*, using both bait and residual film techniques. For the residual film method, the concentrations tested were: for nicotinamide, (0.025, 0.050, 0.075, 0.1, and 0.15%) and (0.02, 0.06, 0.1, 0.14, and 0.18%); for oxamyl, (0.01, 0.03, 0.07, 0.09, and 0.15%) for both species; for imidacloprid, (0.075, 0.15, 0.2, 0.25, and 0.3%) and (0.09, 0.12, 0.15, 0.18, and 0.21%); and for silver nitrate, (0.075, 0.1, 0.15, 0.2, and 0.25%) and (0.08, 0.1, 0.12, 0.14, and 0.16%). The contact technique for testing toxicity followed Ascher and Mirian^45^, where each concentration was diluted with distilled water, and 2 ml was spread on the bottom of Petri dishes by moving the dish gently in circles. The water evaporated, leaving a thin film of the compound. Fifteen snails, split into three replicates of five in three plastic boxes, were used per treatment, with distilled water as a control.

For the poison bait method, the concentrations tested on *T. pisana* and *M. cartusiana* were: for nicotinamide, (0.6, 0.9, 1.2, 1.7, and 1.9%) and (0.9, 1.1, 1.3, 1.5, and 1.7%); for oxamyl, (0.25, 0.5, 1, 1.25, and 1.5%) and (0.01, 0.03, 0.07, 0.09, and 0.15%); for imidacloprid, (1.2, 1.4, 1.6, 1.8, and 2%) and (1.1, 1.3, 1.5, 1.7, and 1.9%); and silver nitrate, (1.1, 1.3, 1.5, 1.7, and 1.9%) and (0.9, 1.1, 1.3, 1.5, and 1.7%). The compounds were mixed with 5 g of sugarcane syrup as an attractant, combined with wheat bran to a total of 100 g, and moistened with small amounts of water to formulate poisonous baits. The control treatment was prepared without adding poisonous baits. Each treatment had 5 g of bait placed in a box with moist soil containing five adult snails. Boxes were covered with muslin netting to prevent escape, and mortality was recorded daily for 7 days.

#### Field experiments

The tested compounds were applied under field conditions in lemon orchards severely infested with *T. pisana* snails, and clover fields infested with *M. cartusiana* at Kafr debe village, Motobas district, Kafr El-Sheikh Governorate, during spring 2023. The tested compounds nicotinamide, imidacloprid, silver nitrate nanoparticles and oxamyl were applied at concentrations of (1.9, 2, 1.9 and 1.5%) and (1.7, 1.9, 1.7 and 1.5%) for *T. pisana* and *M. cartusiana* snails, respectively. The infested area was divided into plots representing the treatments, each of which was about one kirat (175 m^2^) representing the treatments, and each plot was divided into sub-plots representing the replicates. The baits were made by combining the calculated weight of the studied compound with wheat bran as a carrier material (w/w), adding 5% sugarcane syrup as an attractant, and then using the mixture as poison bait. Each week, the baits changed hands. 100 g of prepared baits were placed in each sub-experimental plot. Before treatment and one, three, seven, ten, fifteen and twenty-one days following application, the number of living snails in each plot was counted. Henderson and Tillton’s^46^ formula was used to statistically calculate reduction percentages.

### Molecular diagnosis (RAPD-PCR) or DNA electrophoretic separation (RAPD-PCR)

The randomly amplified polymorphic DNA (RAPD) technique includes the amplification, by polymerase chain reaction (PCR) of random segment of genomic DNA using a single short primer of arbitrary sequence^47^. DNA from the tested two tested snails that treated with different five compounds was extracted according to Agrawal et al.^48^. A set of 30 primers was analyzed based on the accurate amplified bands profiles of DNA fingerprinting and only two different primers were selected because of their good RAPD profiles shown in Table 6.

**Table 6:** The sequence of primers enter the RAPD-PCR reaction.

Primer	Sequence
1	CTG ATA CGC C
2	TGG ACC GGT G

### DNA amplification cycles

The temperature cycling program used with a Perkin-Elmer Gene Amp PCR system (model 2400) was carried out according to Williams et al.^49^ as follows: one cycle at 94 °C for 5 min followed by 30 cycles consisting of one step of denaturation (94 °C) for 40 s, one step of annealing (37 °C) for 1 min, followed by one step of synthesis (72 °C) for 2 min and a final extension step 72 °C for 7 min and finally 4 °C infinitive.

### Band analysis

Reaction products were analyzed by electrophoresis on 1.0% agarose gels stained with ethidium bromide and photographed under UV light. The synthetic DNA, ladder 10,000 bp (Pharmacia) was employed as molecular markers for bands molecular weight. Each amplified band profile was defined by the presence or absence of bands at particular positions on the gel. Profiles were considered different when at least one polymorphic band was identified. Fragments were scored based on standard marker using Gel Analyzer 3 (Egygene) software.

### Data analysis

Using Abbott’s formula, the mortality percentages were calculated and adjusted^50^. In accordance with Finney’s^51^ methodology, the LC_50_ values were calculated using a probit analysis spread sheet calculator^52^. Using Sun’s^53^ methodology, the toxicity index of the substances under investigation was determined as follows: By dividing the LC_50_ of the most effective compounds by the LC_50_ of the tested compounds, and then multiplying the result by 100, the toxicity index is determined.

## Conclusions

The present study demonstrates the varying molluscicidal efficacy of several tested compounds, namely oxamyl, nicotinamide, imidacloprid, and silver nitrate nanoparticles, against two terrestrial snail species, *Theba pisana* and *Monacha cartusiana*, under both laboratory and field conditions. Among all compounds, oxamyl consistently exhibited the highest toxicity and reduction rates, confirming its superior molluscicidal potency. Notably, nicotinamide emerged as a promising alternative, showing considerable bioactivity and field performance, particularly against *T. pisana*, and ranking second to oxamyl in most assessments. Silver nitrate nanoparticles also showed moderate effectiveness, especially under field conditions, while imidacloprid exhibited relatively lower molluscicidal activity. Furthermore, the residual film method proved more efficient than poison bait under laboratory conditions for both snail species. RAPD-PCR molecular analysis revealed significant genetic variations in response to the different treatments, with the highest levels of polymorphism observed in snails treated with oxamyl and nicotinamide, indicating a potential link between compound toxicity and induced genetic alterations. These findings highlight the practical potential of nicotinamide and silver nitrate nanoparticles as environmentally safer alternatives to conventional pesticides, emphasizing their suitability for incorporation into integrated pest management (IPM) strategies. Moreover, monitoring genetic impacts using molecular tools like RAPD-PCR can provide early warning indicators of sublethal effects, supporting more sustainable pest control practices. Future research should investigate the long-term ecological consequences, non-target organism safety, optimal application rates, and cost-effectiveness of these compounds, as well as their integration with biological control agents and cultural practices to enhance sustainable agricultural management.

## Data Availability

The datasets generated during and/or analysed during the current study are available from the corresponding author on reasonable request.
